# Assessment of Knowledge, Attitude, and Practice towards Prevention and Control of Malaria in Halaba Town, Southern Ethiopia, 2017

**DOI:** 10.1155/2021/5665000

**Published:** 2021-08-31

**Authors:** Tadesse Menjetta

**Affiliations:** Hawassa University, College of Health Sciences, Department of Medical Laboratory, P.O. Box 1560, Hawassa, Ethiopia

## Abstract

**Background:**

Malaria is one of the primary public health problems in Ethiopia. Therefore, assessment of situation of the disease and communities' knowledge and perceptions about this disease is necessary to introduce appropriate preventive and control measures. Hence, this study was aimed to assess the knowledge, attitude, and practice towards malaria in Halaba town, SNNPR, Ethiopia.

**Methods:**

A community-based cross-sectional study was conducted in Halaba town from June 2017 to September 2017. A multistage random sampling technique was used to select the study participants. A total of 421 were interviewed to assess their knowledge, attitude, and practice towards malaria.

**Results:**

About 280 (66.5%) respondents had ever heard of malaria. Most of the respondents (63.4%) attributed the cause of malaria to mosquito bites. However, some of the respondents (36.6%) mentioned contact with malaria patients, lack of personal hygiene, staying together, and transmission via breathing as the causes of malaria. Sleeping under mosquito nets, draining stagnant water, and indoor residual spraying were the most frequently mentioned malaria preventive measures perceived and practiced by the respondents.

**Conclusions:**

A high level of knowledge about the cause, transmission, and preventive methods of malaria was detected among the community in Halaba town. However, a significant proportion had misconceptions about the cause and transmission of malaria suggesting the necessity of health education to raise the community's awareness about the disease.

## 1. Introduction

Malaria is an infectious vector-borne disease caused by four common species of protozoan parasites of the genus Plasmodium: *Plasmodium falciparum, Plasmodium vivax*, *Plasmodium ovale,* and *Plasmodium malariae*. [1] It is still one of the worldwide public health problems. In 2010, globally, the disease caused 216 million cases and 655,000 deaths, of which 81% of the cases and 91% of the deaths were from Sub-Saharan Africa [2]. According to WHO 2012 report, malaria is the most important tropical parasitic disease and kills more people than any other communicable diseases, except tuberculosis [3].

In Ethiopia, more than 3/4 of the landmass is malarious and about 68% of people of the total population are residing in areas at risk of malaria infection [4]. Despite significant improvements in prevention and control in the past decades, malaria remains a significant public health concern in Ethiopia. Currently, a range of effective malaria control interventions are being scaled up in Ethiopia to improve access and equity to preventive as well as curative health services. These initiatives include prompt and effective treatment of malaria, selective vector control including insecticide-treated nets (ITNs) and indoor residual spraying (IRS), and prevention and control of the epidemic [5].

Even though malaria primarily occurs in rural areas [6], studies are reporting increased malaria transmission in urban areas [7]. The reason may be linked to the rapid urbanization with a lack of proper sanitation and poor drainage of water that may favor malaria transmission [8].

Addressing community knowledge, attitudes, and practices can be of critical importance towards developing malaria control strategies. There is still a lack of studies concerning knowledge, attitudes, and practices in Ethiopia. Thus, this study is intended to determine the knowledge, attitude, and practices towards malaria in Halaba town, Southern Ethiopia.

## 2. Methodology

### 2.1. Study Area

A community-based cross-sectional household survey was conducted in Halaba town from June 2017 to September 2017. Halaba town is located 315 km from Addis Ababa, the capital of Ethiopia, and 92 km from Hawassa, the capital city of the region. It is situated at an altitude of 1554–2149 meters above sea level. The weather condition is categorized as kola (71.66%), woynadega (28.17%), and dega (0.17%).

### 2.2. Study Design

This study was a community-based cross-sectional household survey.

### 2.3. Sample Size and Sampling Procedure

The sample size for the study was calculated using single population proportion formula (*n*=(*Zα*/2)^2^*p*((1 − *p*)/*d*^2^)) at 95% confidence interval (CI) (*Zα*/2 = 1.96), with 5% margin of error [9]. Using P to be 47% from a previous study [10], and including a 10% nonresponse rate, the final sample size became 421. A total of 421 households were selected using a probability proportion to the size of households in five “Kebeles” (the smallest administrative units) of the town. The shared households for each Kebele were divided by the total number of households in a given Kebele to determine a sampling interval for selected households. Accordingly, every 5^th^ households were selected using a systematic random sampling technique. The study population included those aged 18 years and above of both sexes.

### 2.4. Data Collection and Quality Control

A standard structured questionnaire was designed based on the study objective to collect information regarding socio-demographics and KAP of the study participants about malaria. The questionnaires were pretested in nonselected Kebeles by a pilot study for assessing content validity, appropriateness, and question comprehensibility. Three nurses from health centers in the study area were selected to collect data. Training was given to the data collectors for three days on how to conduct the interview, content of the questionnaire, data quality, and ways to approach respondents. After the data collection process, the data were rechecked and cleaned.

### 2.5. Data Analysis Plan

Data were double entered and cross-checked using EpiData version 3.1 and analyzed using SPSS version 20.

### 2.6. Ethical Consideration

The study obtained ethical clearance from the Institutional Review Board (IRB) of Hawassa College of Health Sciences. A supportive letter was obtained from the Halaba town health office before data collection and written informed consent was obtained from the participants.

## 3. Results

### 3.1. Socio-Demographic Characteristics of Study Participants

The study enrolled a total of 421 households. Of these, 248 (58.9%) were male and 173 (41.1%) were female. The mean age of the study participants was 28.3 years. About 82% were married, and the majority of respondents attended grades 1–8 (Table 1).

### 3.2. Knowledge and Attitude of Household Respondents towards Malaria

About 280 (66.5%) of the participants knew that malaria is a transmittable disease. The majority of them, 267 (63.4%), believed that mosquitoes are responsible for the transmission. However, 154 (36.6%) of respondents associated the cause of malaria with contact with malaria patients, lack of personal hygiene, staying together, and transmission via breathing.

About 79.1% of the study participants mentioned stagnant water as breeding site for mosquitoes, whereas others responded running water 6.9%, did not know 7.6%, and other sites 6.4% as a breeding site. About 82.7% had reported mosquito biting on human beings at night time (Table 2).

All respondents (421) had ever heard of malaria. Regarding the source of information, about 370 (87.9%) of the respondents got the information from health institutions by health extension workers. About 30 (7.1%) of the respondents got information via mass media. Fever and headache were frequently mentioned symptoms reported by 209 (49.6%) and 172 (40.8%), respectively (Table 3).

About 80% of participants responded that under-five children are more affected, whereas about 15% mentioned that pregnant mothers are more affected than any other group by malaria. About 256 (60.8%) of the respondents have noticed stagnant water in their surroundings, but only 154 (60%) of them have tried to drain it. The main reasons they mentioned for not draining stagnant water were not knowing the negative consequences of stagnant water 70 (68.8%), lack of awareness 19 (18.8%), and lack of time 13 (12.4%).

### 3.3. Malaria Prevention Practices Used by the Study Respondents

The majority of the respondents, 409 (97.1%), reported to have ever used the bed net and 311 (76%) of them are still using it. Those who are not currently using bed net, 98 (24%), mentioned their reason for not using it is due to lack of awareness 44 (44.9%), fear of side effects 14 (14.3%), and lack of access 40 (40.8%). Regarding the priority who to use bed net, 291 (69.1%) believed that under-5 children should get priority to use bed net, while 65 (15.4%) responded with the priority for pregnant mother. The majority of the respondents, 391 (92.9%), had a practice of DDT spraying at their home while 30 (7.1%) never sprayed their home. The reasons that respondents mentioned for not using DDT in their home were lack of access 23 (76.7%), lack of awareness 3 (10%), and fear of side effects 4 (13.3%).

## 4. Discussion

A community-based cross-sectional study on communities' knowledge, attitude, and practices towards the cause, transmission, prevention, and control measures of the disease was undertaken in Halaba town, Southern Ethiopia. The results showed that there is high awareness about malaria among the communities of Halaba town.

The study results showed that all respondents had ever heard of malaria which is higher than the study done in Amhara National Regional State which shows 87% of respondents heard about malaria [10]. About 85% of them believed that mosquito bites were responsible for the transmission of the disease, which is comparable with a study conducted in Gurage Zone (86.2%) and Gamo Gofa Zone (83.7%), Southern Ethiopia [8, 9]. However, it was higher than a study reported in Jimma town (71.8%), Ethiopia (10). This awareness is lower than the level reported in Arba Minch (98.2%), Shewa Robit (95.6%), Ethiopia [11, 12]. The difference might be due to variation in accessibility of health extension services, behavioral change, environmental variation, type of study population, and level of endemicity in the study areas.

Although the majority of the respondents associated the cause of malaria with mosquito bites, about 36.6% of respondents mentioned contact with malaria patients, lack of personal hygiene, staying together, and transmission via breathing as the causes of malaria. Such misunderstandings have also been in Shewa Robit town and Assosa Zone, Ethiopia [12, 13]. Despite these facts, the perception of these by the study participants as direct causes of malaria may influence the actual prevention mechanism they may choose. Thus, this should be corrected with appropriate health education which could change their behavior. Studies have reported a better understanding of the causes of malaria in communities who had better awareness about the issue through health education [14, 15].

Most of the respondents were also familiar with at least one of the classical symptoms of malaria which is expected for a population in endemic areas where people are aware of the clinical manifestations of the disease. In this study, fever (49.6%) and headache (40.8%) were the most common signs and symptoms of malaria mentioned by the participants. This finding is lower when compared to the findings from Areka town (93.6%) and Pawe District (83%) in Ethiopia [16, 17].

In this study, about 82.7% of the study participants responded that mosquitoes bite humans during the night. It was comparable with the study reported in Shewa Robit (83.8%) [12]. However, it was higher than the study reported in Gamo Gofa Zone (61.7%), Southern Ethiopia [18], and Amhara National Regional State (48%), Northern Ethiopia [19]. This high perception among respondents of the present study is encouraging to take appropriate preventive measures and proper use of mosquito nets.

In this study, all respondents believed that malaria is a preventable disease which is in agreement with other studies conducted in Ethiopia reporting higher knowledge of communities on malaria as preventable [20].

Regarding mosquito breeding sites, 79.1% of respondents claimed that stagnant water was the main site that is higher than results from Gamo Gofa Zone (34.0%) [18] and Amhara National Regional State (72.6%) [10], Northern Ethiopia.

Even though the majority of the respondents (97.1%) reported to have ever used a bed net, currently there are about 24% who were not frequently using it. Such misuse was reported in Tanzania [21]. Use of mosquito nets, filling and draining mosquito breeding sites (stagnant and surface water), and house spray with insecticides were the three main types of malaria preventive measures frequently reported by the current study participants. This agrees with previous observations from other parts of Ethiopia, Tanzania, and Bangladesh [21–23]. In the current study, 92.9% of respondents sprayed their houses with DDT which is higher than the study conducted in Shewa Robit, Ethiopia, where 70.4% reported [12].

### 4.1. Limitations

The limitations of this study include failure to have direct observation of ITNs usage and performing a parasitological survey.

## 5. Conclusions and Recommendations

A high level of knowledge about the cause, transmission, and preventive methods of malaria was detected among the community in Halaba town. However, a significant proportion had a misconception about the cause and transmission of malaria, suggesting the necessity of health education to raise the community's awareness about the disease.

## Figures and Tables

**Table 1:** Socio-demographic characteristics of the study participants, Halaba town, Southern Ethiopia, 2017.

Variable	Category	*n* (%)
Residence	Rural	279 (66.3)
	Urban	142 (33.7)

Age	18–24	155 (36.8)
	25–34	172 (40.9)
	35–44	69 (16.4)
	45–54	21 (5.0)
	55 and above	4 (1.0)

Sex	Male	248 (58.9)
	Female	173 (41.1)

Marital status	Married	345 (81.9)
	Single	45 (10.7)
	Divorced	25 (5.9)
	Widowed	6 (1.4)

Educational status	Illiterate	121 (28.7)
	Read and write	9 (2.1)
	Grade 1–8	228 (54.2)
	Grade 9–12	60 (14.3)
	>12^th^ grade	3 (0.7)

Occupational status	Farmer	159 (37.8)
	House wife	146 (34.7)
	Merchant	83 (19.7)
	Government employed	6 (1.4)
	Others	27 (6.4)

Income of the respondents	<500 birr per month	172 (40.9)
	500–1000 birr per month	179 (42.5)
	1001–1500 birr per month	33 (7.8)
	1501–2000 birr per month	34 (8.1)
	>2000 birr per month	3 (0.7)

**Table 2:** Knowledge and attitude of respondents regarding mosquito breeding site, biting time, and transmission of malaria in Halaba town, Southern Ethiopia, September 2017.

Variable		Number	Percent
Mosquitoes breeding site	Stagnant water	333	79.1
	Running water	29	6.9
	Do not know	32	7.6
	Others	27	6.4

Mosquito biting time	At night	348	82.7
	At day	43	10.2
	At any time	15	3.6
	Do not know	15	3.6

Means of transmission	Mosquito bite	267	63.4
	Staying together	34	8.1
	Lack of personal hygiene	41	9.7
	By breathing	35	8.3
	Contact with malaria patient	29	6.9
	Others	15	3.6

**Table 3:** Knowledge of respondents about symptoms of malaria in Halaba town, Southern Ethiopia, September 2017.

Variable	Number	%
Fever	209	49.6
Headache	172	40.8
Chills	113	26.8
Loss of appetite	55	13
Joint pain	51	12.1
Others	7	1.6

## Data Availability

The datasets used and/or analyzed during the current study are included in the article.
